# Psoriasis and Bullous Pemphigoid: An Updated Review of Clinical Association, Targeted Therapy Exposures and Therapeutic Implications

**DOI:** 10.3390/biom16101391

**Published:** 2026-09-24

**Authors:** Shengnan Cui, Bingjie Zhang, Li Li

**Affiliations:** Department of Dermatology, State Key Laboratory of Complex Severe and Rare Diseases, Peking Union Medical College Hospital, Chinese Academy of Medical Sciences and Peking Union Medical College, Beijing 100730, China; b2024001195@student.pumc.edu.cn (S.C.); zhangbingjie@pumch.cn (B.Z.)

**Keywords:** psoriasis, bullous pemphigoid, IL-23/IL-17 axis, biologics, Janus kinase inhibitors, paradoxical reaction

## Abstract

Psoriasis is a chronic immune-mediated inflammatory dermatosis driven predominantly by dysregulation of the interleukin-23–T helper 17 axis. Bullous pemphigoid is a distinct autoimmune subepidermal blistering disorder characterized by autoantibody-mediated injury at the dermal–epidermal junction and a T helper 2-polarized, eosinophil-rich inflammatory response. Despite their marked clinical and immunopathological differences, accumulating epidemiological evidence suggests that the coexistence of psoriasis and bullous pemphigoid is unlikely to be incidental. Therapeutic exposure may also contribute to this association. Biologic agents used to treat psoriasis, and in selected cases bullous pemphigoid, have occasionally been reported in temporal association with new-onset bullous pemphigoid. These observations raise the hypothesis that divergent treatment-related effects may partly reflect differences in the prevailing immune milieu rather than a uniform property of a specific drug class. This review summarizes current evidence on the clinical association between psoriasis and bullous pemphigoid, critically examines targeted therapy exposures and their divergent clinical contexts, and discusses the immunopathogenic mechanisms that may contribute to the development of bullous pemphigoid in patients with psoriasis. Particular attention is given to the implications of these findings for individualized clinical management.

## 1. Introduction

Psoriasis is a chronic, immune-mediated inflammatory disease affecting approximately 0.5–3% globally, with marked geographic variation [1]. Its pathogenesis involves genetic susceptibility, particularly HLA-C*06:02, together with immune dysregulation centered on the interleukin-23–T helper 17 (IL-23–Th17) axis and environmental triggers such as infections, obesity, and smoking. These factors collectively sustain systemic inflammation and contribute to the development of various autoimmune and inflammatory comorbidities [2]. Clinically, psoriasis comprises several heterogeneous subtypes, of which plaque psoriasis is the most common, whereas guttate, pustular, and erythrodermic forms are less frequent. Management is tailored according to disease severity, with topical corticosteroids and vitamin D analogs used for mild disease, and systemic agents or biologic therapies targeting tumor necrosis factor-α (TNF-α), interleukin-17 (IL-17), and interleukin-23 (IL-23) used for moderate-to-severe cases.

Bullous pemphigoid (BP) is the most common autoimmune subepidermal blistering disease and typically affects older adults. Recent studies suggest that the incidence of BP ranges from 2 to 30 new cases per million people per year, with marked geographic and methodological heterogeneity, and appears to have increased in recent years. The incidence also rises markedly with age, reaching 190–312 new cases per million people per year among individuals older than 80 years [3]. Its pathogenesis involves autoantibodies directed against the hemidesmosomal proteins bullous pemphigoid antigen 180 (BP180; type XVII collagen) and bullous pemphigoid antigen 230 (BP230), leading to complement activation, inflammatory cell recruitment, and subepidermal blister formation [4]. Clinically, BP is characterized by widespread tense bullae, often preceded by pruritic urticarial or eczematous lesions, with mucosal involvement observed in a subset of patients. Treatment depends on disease severity and ranges from topical corticosteroids to systemic immunosuppressive agents and biologics, with particular emphasis on balancing efficacy and safety in older patients.

Epidemiological evidence supports an association between psoriasis and bullous pemphigoid despite their distinct clinical manifestations and immunopathological profiles. This relationship may reflect the interplay of chronic cutaneous inflammation, immune dysregulation, basement membrane zone injury, and therapeutic exposures, including biologic agents and small-molecule inhibitors. However, the mechanisms underlying this comorbidity remain incompletely understood, and interpretation of the available clinical evidence is complicated by the predominance of isolated case reports and small case series.

Although the association between psoriasis and bullous pemphigoid has been reviewed previously, recent developments warrant an updated synthesis. The expanding use of biologic and small-molecule targeted therapies has introduced new clinical scenarios in which the same therapeutic pathway has been reported in association with either disease control or presumed disease activation. In addition, recently published cases include newer therapeutic agents, such as IL-23p19 inhibitors, JAK inhibitors, PDE4 inhibitors, IL-36R blockade, and IL-4Rα blockade, which were incompletely represented in earlier reviews. Interpretation of these reports is further complicated by the coexistence of therapeutic use and temporally associated disease onset or exacerbation, precluding simple causal interpretation.

Accordingly, this updated narrative review focuses on the targeted therapy era and summarizes the literature published through 14 April 2026. In addition to reviewing the epidemiological evidence and proposed pathogenic mechanisms, we provide a patient-level synthesis of 41 published cases involving targeted therapy exposure. These cases are classified descriptively as temporal association, therapeutic use, or overlapping exposure to facilitate clinical interpretation rather than to infer causality. We hope this framework will assist clinicians in selecting targeted therapies for patients with concomitant psoriasis and bullous pemphigoid while recognizing that the current evidence does not support conclusions regarding incidence, comparative safety, efficacy, or drug-specific causality.

## 2. Literature Search and Study Selection

Consistent with the narrative scope of this review, the literature search was organized into three complementary streams addressing: (1) epidemiological evidence on the association between psoriasis and bullous pemphigoid; (2) studies relevant to their potential immunopathological links; and (3) patient-level reports involving targeted therapy exposure. PubMed/MEDLINE, Web of Science Core Collection, and Google Scholar were searched. During revision, the searches were rerun on 2 September 2026 for reproducibility verification while retaining the prespecified publication cutoff of 14 April 2026. Targeted-therapy terms were not required for the epidemiological or mechanistic searches. Complete database-specific search strategies and retrieved record counts are provided in Supplementary Table S1.

For the patient-level targeted-therapy synthesis, a broad disease-based search was used to minimize the possibility of missing relevant reports in which the therapeutic exposure was not specified in the title or abstract. The search identified 459 records in PubMed/MEDLINE and 463 records in Web of Science Core Collection; the first 200 relevance-ranked Google Scholar records were additionally screened. Across these sources, 1122 records were identified. After removal of 449 exact duplicates based on DOI, PMID, or normalized article title, 673 unique records remained. The final synthesis included 36 publications describing 41 individual patients.

Original case reports, case series, observational studies, and reviews were considered when they addressed clinical, epidemiological, mechanistic, or therapeutic aspects of the psoriasis–BP association. For the case summary table, reports were included only when individual patient-level data were available. Extracted variables included disease chronology, targeted therapy exposure, clinical manifestations, latency, previous BP history, diagnostic evidence, dechallenge and rechallenge information, alternative potential triggers, concomitant treatments, treatment course, follow-up, and outcomes. Studies were excluded if they did not involve both psoriasis and BP, lacked sufficient clinical information, or described other autoimmune blistering diseases without an adequately supported diagnosis of BP. For the patient-level synthesis, BP was considered adequately documented when the original report explicitly diagnosed BP and provided compatible clinical findings together with objective diagnostic support, including histopathology, direct or indirect immunofluorescence, salt-split skin findings, and/or BP180/BP230 serology. DIF or BP180/BP230 positivity was not required in every case because the diagnostic work-up varied across reports. For patients with previously established BP, the documented prior diagnosis was accepted when the complete original diagnostic work-up was not reproduced in the subsequent treatment report. Cases with an alternative or overlapping pemphigoid diagnosis that could not be reasonably classified as BP were excluded from the BP-specific patient-level synthesis. Only English-language publications were included to ensure reliable extraction of case-level data.

Targeted therapy exposure was classified descriptively according to the clinical context reported in each original publication. Cases were categorized as temporal association when initiation of a targeted therapy preceded new-onset or worsening psoriasis or BP, as therapeutic use when the therapy was administered primarily for disease control, and as overlapping exposure when treatment occurred during the disease course without a sufficiently clear temporal or mechanistic relationship to disease onset or exacerbation. These categories were used for descriptive purposes only and were not intended as formal causality assessments. To reduce reliance on subjective attribution, objective causality-related features were extracted separately when reported, including latency, previous BP history, dechallenge and rechallenge responses, alternative potential triggers, concomitant treatments, and the diagnostic evidence supporting BP. For cases in which BP developed or worsened after treatment exposure, causal support was further graded as strong, moderate, or weak/uncertain. Strong support required a positive rechallenge. Moderate support required BP onset or exacerbation after treatment initiation, a compatible reported latency, improvement after treatment withdrawal, and no compelling alternative cause. Cases with temporal association alone, unclear chronology, confounded dechallenge, or plausible alternative causes were classified as weak/uncertain. No fixed latency threshold was applied. Missing information was recorded as NR and was not inferred. Formal causality tools, including the Naranjo scale and the World Health Organization–Uppsala Monitoring Centre system, were not applied because most reports lacked the standardized information required for reliable assessment and frequently involved concomitant treatments or plausible alternative explanations.

Potentially overlapping cases were assessed by comparing patient demographics, disease chronology, targeted agents, exposure timing, clinical features, and publication details. When duplicate reporting was suspected, the report with the most complete patient-level information was retained; when completeness was comparable, the more recent publication was used. Given the narrative scope of this updated review, no formal risk-of-bias assessment or quantitative synthesis was performed.

## 3. Clinical and Immunologic Links Between Psoriasis and Bullous Pemphigoid

### 3.1. Epidemiological Association and Methodological Considerations

The coexistence of psoriasis and autoimmune bullous diseases was first documented in 1929. The specific association between psoriasis and BP was subsequently examined in a controlled study by Grattan et al., who reported a significantly higher prevalence of psoriasis among patients with pemphigoid than in the general population (*p* < 0.01, Poisson distribution) [5]. This observation has since been consistently supported by subsequent epidemiological studies [6,7,8]. A meta-analysis published by Phan et al. confirmed that psoriasis was significantly more common in patients with BP than in controls (2.6% vs. 1.1%; odds ratio [OR], 2.5; 95% confidence interval [CI], 1.4–4.6) [9]. Consistent findings were reported in a large population-based study by Kridin et al., which demonstrated a bidirectional association between psoriasis and BP. BP was associated with an increased risk of subsequent psoriasis (adjusted hazard ratio, 2.60; 95% CI, 1.59–4.27), whereas a history of psoriasis was associated with an increased risk of BP (adjusted OR, 1.53; 95% CI, 1.14–2.06) [10]. More recently, a nationwide Korean case–control study further strengthened this epidemiological evidence by showing that psoriasis was significantly more prevalent among patients with BP than among matched controls during the five years preceding BP diagnosis (4.93% vs. 1.62%; OR, 3.15; 95% CI, 2.58–3.85) [11]. Beyond observational evidence, two bidirectional Mendelian randomization studies suggested that genetically predicted psoriasis was associated with a modestly increased risk of BP (inverse-variance weighted OR, approximately 1.26), whereas genetically predicted BP was not associated with an increased risk of psoriasis [12,13]. Taken together, observational studies support a bidirectional epidemiological association, whereas current Mendelian-randomization evidence suggests asymmetric genetic susceptibility, with support for psoriasis increasing susceptibility to BP but not for the reverse direction.

Although the association between psoriasis and BP has been reported across different populations, evidence for causality remains limited. Most studies are retrospective case–control, registry-based, or administrative database analyses, with substantial variation in case ascertainment, eligibility criteria, covariate adjustment, and follow-up. Age is an important source of residual confounding because BP incidence increases markedly in older populations and age-related comorbidities are common. Treatment exposure is another concern, as phototherapy and systemic or biologic therapies are not consistently captured and may confound or modify the observed association. Surveillance bias is also possible because patients with psoriasis are more likely to receive regular dermatological care, increasing the likelihood that pruritic or blistering disorders are recognized. Thus, the available epidemiological evidence supports an association between psoriasis and BP but is insufficient to establish a direct causal relationship. Key epidemiological studies and their methodological limitations are summarized in Table 1.

### 3.2. Clinical and Immunopathological Characteristics

Clinically, patients with concomitant psoriasis and BP often show a pattern in which psoriasis precedes the onset of BP by many years. In a hospital-based case–control study, psoriasis preceded BP in all patients with both conditions, with a mean interval of 25.2 ± 18.6 years, whereas in a large population-based study the mean latency was 11.8 ± 10.7 years when BP developed after psoriasis [10,14]. Nevertheless, population-based data also suggest that BP may develop relatively early in a subset of patients. In a nationwide Taiwanese cohort, the mean interval between psoriasis and BP was 2.86 years, with the highest incidence observed during the first year after psoriasis diagnosis [7]. These findings indicate substantial heterogeneity in disease chronology and suggest that the interval between the two disorders may vary according to patient characteristics or underlying pathogenic mechanisms.

Compared with classic BP, psoriasis-associated BP appears to exhibit a distinct clinical phenotype, although the available evidence remains somewhat inconsistent. In the population-based study by Kridin et al., patients with concomitant disease were more frequently male than those with isolated BP (53.5% vs. 42.2%, *p* = 0.023) and developed BP at a younger age (74.4 ± 11.9 vs. 76.8 ± 14.4 years, *p* = 0.049) [10]. Comparable findings were reported in a hospital-based case–control study, in which patients with both diseases presented at a younger age than those with isolated BP (68.5 ± 13.7 vs. 78.0 ± 11.9 years, *p* = 0.003). Mucosal involvement also appeared to be more common in patients with concomitant disease (33.3% vs. 15.4%), although the difference did not reach statistical significance (*p* = 0.077) [14]. In addition, coexisting psoriasis was associated with more prolonged corticosteroid treatment, reflected by higher rates of long-term systemic corticosteroid use (74.3% vs. 64.8%, *p* = 0.048) and long-term topical corticosteroid use (99.0% vs. 94.0%, *p* = 0.035) [10].

Available evidence does not identify a consistent histopathological pattern that distinguishes psoriasis-associated BP from idiopathic BP. In a retrospective comparative study, the evaluated histopathological features did not differ significantly between the two groups, although neutrophil-predominant inflammation was observed in some patients with concomitant psoriasis [15]. Immunopathological differences have been reported in individual cohorts. One study found lower serum anti-BP180 NC16A levels, more frequent isolated linear complement component 3 (C3) deposition, and less frequent linear immunoglobulin G (IgG) deposition along the basement membrane zone in patients with concomitant psoriasis than in those with BP alone [16]. These findings have not been consistently reproduced and should be considered potential phenotypic variations rather than defining features of psoriasis-associated BP.

Patients with psoriasis-associated BP often have long-standing psoriasis and previous exposure to phototherapy, systemic therapies, or biologics. In a Taiwanese nationwide cohort, 20% of patients who subsequently developed BP had received phototherapy, while individual reports have documented prior exposure to methotrexate, azathioprine, adalimumab, and ustekinumab [7,17,18]. These treatment histories do not establish causality and may instead reflect cumulative treatment exposure or greater psoriasis severity. Overall, selected clinical and immunopathological differences between psoriasis-associated and idiopathic BP have been reported, but the evidence remains limited and heterogeneous. The main reported features are summarized in Table 2.

### 3.3. Diagnostic Considerations and Differential Diagnosis

An important differential diagnosis in patients with psoriasis who develop a subepidermal blistering eruption is anti-p200 pemphigoid, which can closely resemble BP both clinically and histopathologically and is strongly associated with psoriasis. In a systematic review of 113 patients with anti-p200 pemphigoid, 28.3% had concomitant psoriasis and 66.1% showed clinical features resembling BP [19]. Terminology in this area requires caution. Although laminin γ1 was identified as a major target antigen, antibodies against recombinant laminin γ1 are detected in only a subset of patients, and laminin β4 has more recently been identified as another autoantigen [19,20].

Diagnosis requires integration of clinical findings, histopathology, immunofluorescence, and serological testing. Direct immunofluorescence of perilesional skin is central to the diagnosis of BP and typically shows linear IgG and/or C3 deposition along the basement membrane zone. A similar pattern, however, can occur in anti-p200 pemphigoid and does not reliably distinguish the two diseases [19,21]. Indirect immunofluorescence on 1 M NaCl salt-split human skin provides additional discriminatory information: circulating IgG in BP most commonly binds to the epidermal side of the split, whereas anti-p200 pemphigoid typically shows dermal-side binding. Because dermal-side reactivity is not specific to anti-p200 pemphigoid, other subepidermal autoimmune blistering diseases should be excluded and, where available, antigen-specific testing performed. Serum anti-BP180 IgG, followed when appropriate by anti-BP230 IgG, can further support a diagnosis of BP, although negative serology does not exclude it [21]. Anti-p200 pemphigoid should therefore be considered in patients with psoriasis who have an atypical serological profile or dermal-side binding on salt-split skin [22].

### 3.4. Immunological Overlap and Immune Plasticity

The coexistence of psoriasis and BP may reflect convergent immune mechanisms rather than shared genetic susceptibility alone. Psoriasis is predominantly characterized by Th17-driven inflammation, whereas BP is associated with a Th2-polarized, eosinophil-rich inflammatory response. This immunological contrast has led to the hypothesis that the two diseases may represent different states along a continuum of immune polarization. However, direct evidence supporting a sequential transition from Th17- to Th2-dominant immunity within individual patients remains limited [23,24]. A seminal study by Yasukawa et al. described the development of generalized pustular psoriasis during corticosteroid treatment for BP and demonstrated dynamic changes in Th1-, Th2-, regulatory T cell (Treg)-, and Th17-related immune markers across different stages of disease progression [25]. These observations are consistent with the concept of immune plasticity but do not support a uniform or unidirectional transition from psoriasis to BP. A cross-disease single-cell transcriptomic study placed several inflammatory dermatoses within a broader Th2/Th17 transcriptional framework and included both psoriasis and BP; however, BP was represented by only one sample, limiting disease-specific inference [26]. Together, these findings are compatible with dynamic immune adaptation but do not establish a specific or directional shift in immune polarization in individual patients. The conventional distinction between Th17-dominant psoriasis and Th2-biased BP is useful but does not fully capture the immunological complexity of BP. Its pathogenesis involves type 2 immunity, IL-17/IL-23 signaling, neutrophils, complement, and regulatory immune pathways. Increased IL-17 and IL-23 activity has been reported in BP, and neutrophil extracellular traps and granulocyte-derived proteases may contribute to dermal–epidermal injury [27,28,29]. Complement activation is also a well-established component of BP pathogenesis [4]. Experimental evidence further suggests that regulatory T-cell dysfunction may impair tolerance to BP-associated antigens and facilitate autoreactive humoral responses [30,31]. BP therefore extends beyond a simple Th2-dominant model. Whether these pathways specifically contribute to the association between psoriasis and BP, however, remains unclear.

It is important to distinguish mechanisms established in BP from those proposed to explain its association with psoriasis. Autoantibody-mediated injury, complement-dependent inflammation, and granulocyte-derived proteolytic activity are well-established features of BP pathogenesis. By contrast, the proposed sequence whereby chronic psoriatic inflammation remodels the basement membrane zone, exposes cryptic BP antigens, and promotes epitope spreading has not been prospectively demonstrated in patients with psoriasis. BMZ remodeling and epitope spreading should therefore be regarded as biologically plausible linking mechanisms rather than established steps in the progression from psoriasis to BP.

### 3.5. Proposed Mechanistic Links Between Psoriasis and Bullous Pemphigoid

The coexistence of psoriasis and BP may reflect shared inflammatory susceptibility together with environmental and treatment-related exposures, although the causal contribution of these factors remains uncertain. Previous studies have implicated several anti-psoriatic interventions as potential contributors to BP development, including phototherapy, topical agents such as corticosteroids, tar, salicylic acid, and anthralin, as well as biologic therapies [32]. Among these factors, phototherapy has been most consistently associated with subsequent BP onset. Ultraviolet radiation has been proposed to induce epidermal injury and alter the antigenicity of the basement membrane zone, thereby exposing or modifying autoantigens and facilitating the formation of autoantibodies against BP-associated antigens [33]. Although this mechanism remains hypothetical, it provides a biologically plausible explanation for the reported association. Other reported associations are less well established. Several case reports have described possible drug-associated cases involving agents such as losartan. Infectious exposures have also been implicated, with streptococcal infection, hepatitis C, and coronavirus disease 2019 reported in temporal association with BP onset in individual patients [23,34,35,36]. However, the available evidence is largely limited to isolated case reports, and these associations have yet to be confirmed in larger epidemiological studies.

Basement membrane zone (BMZ) alterations and epitope spreading are among the most widely proposed mechanisms linking psoriasis and BP, although their contribution to psoriasis-associated BP remains unproven [10]. Structural changes at the dermal–epidermal junction have been demonstrated in psoriatic skin, while epitope spreading has been documented during BP progression. Chronic psoriatic inflammation may impair BMZ integrity in both lesional and clinically uninvolved skin through alterations in laminin networks and α5β1 integrin expression, potentially increasing antigen accessibility and exposing cryptic epitopes. This may facilitate intramolecular spreading within BP180 or intermolecular spreading to related autoantigens, including BP230 and laminin-332 [6]. An alternative pattern of autoantigen recognition has also been reported in long-standing psoriasis preceding BP, with preferential reactivity to linear IgA disease antigen 1 (LAD-1) rather than the non-collagenous 16A (NC16A) domain of BP180 [37]. This finding suggests that autoantibody specificity may be shaped by the cumulative inflammatory history of the skin. Innate immune activation may further amplify tissue injury, as neutrophil-derived proteases and neutrophil extracellular traps can promote BMZ damage, antigen exposure, and complement activation [15,38]. The temporal sequence and causal contribution of these processes to psoriasis-associated BP remain uncertain.

Convergent cytokine networks may represent an additional mechanistic link between psoriasis and BP [39]. Both diseases are characterized by increased IL-1β expression and activation of the IL-23/IL-17 axis, which may promote keratinocyte activation, neutrophil recruitment, proteolytic activity, and tissue remodeling. The enrichment of IL-17^+^ cluster of differentiation 4-positive T cells in BP lesions further reflects immunological features shared with psoriasis. Within this inflammatory milieu, persistent immune activation or therapeutic perturbation may favor the development of Th2-polarized, autoantibody-mediated inflammation in susceptible individuals. However, direct evidence demonstrating that these cytokine changes drive class-switch recombination or pathogenic autoantibody production specifically in psoriasis-associated BP remains lacking. Cytotoxic effector pathways may further contribute to tissue injury. Granzyme B, released by cytotoxic T cells and natural killer cells, has been implicated in autoimmune skin inflammation and may promote BMZ damage, although its specific role in psoriasis-associated BP remains hypothetical [40]. Overall, BMZ disruption, epitope spreading, innate immune activation, convergent cytokine signaling, immune plasticity, and cytotoxic effector pathways are unlikely to represent independent mechanisms. Instead, they probably constitute an interconnected pathogenic network whose relative contribution varies across disease stages and individual patients. Defining the temporal and mechanistic relationships among these processes remains an important challenge for future studies (Figure 1).

## 4. Targeted Therapy Exposures and Clinical Associations

Biologic and small-molecule targeted therapies have become integral to the management of psoriasis and, in selected cases, concomitant BP. However, published reports have also described new-onset or worsening BP in temporal association with targeted therapy exposure in patients with psoriasis. These observations should be interpreted cautiously because the available evidence is derived predominantly from case reports and does not establish a consistent causal relationship for any therapeutic class. To better characterize this emerging clinical scenario, we reviewed published cases involving targeted therapy exposure in patients with concomitant psoriasis and BP, with the aim of summarizing exposure patterns, presumed roles, and therapeutic outcomes.

As of 14 April 2026, a total of 41 individual cases of concomitant psoriasis and BP associated with targeted therapy exposure were identified. A concise summary by therapeutic class, including the underlying evidence type, is provided in Table 3, while detailed patient-level information is presented in Supplementary Table S2. IL-17 inhibitors were the most frequently represented therapeutic class in the published patient-level literature (17 cases), followed by IL-12/23 or IL-23 inhibitors (13 cases), JAK inhibitors (4 cases), TNF-α inhibitors (3 cases), PDE4 inhibitors (2 cases), and other targeted therapies, including spesolimab and dupilumab (2 cases). Fifteen cases showed a temporal association with BP onset or exacerbation, most commonly involving IL-17 or IL-23 blockade. In the remaining cases, these agents were used to treat psoriasis or represented overlapping exposures without a clear temporal or mechanistic relationship to BP onset. These findings should therefore be interpreted as a descriptive summary of published cases rather than evidence of incidence, relative risk, or drug safety, given the inherent influence of publication bias, incomplete reporting, and confounding by indication. The hierarchy of evidence should be considered when interpreting these reports. Evidence for individual drugs in psoriasis-associated BP is derived largely from case reports and small case series. Systematic reviews can summarize published cases but lack exposure denominators and therefore cannot estimate incidence or compare risk across therapeutic classes. Pharmacovigilance studies may identify broader safety signals for psoriasis biologics, but spontaneous reporting is limited by reporting bias, incomplete clinical information, and the absence of reliable exposure denominators. Such data are useful for signal detection but cannot establish causality. Accordingly, differences in the number of published BP cases across therapeutic classes should not be interpreted as differences in BP risk. Latency between initiation of targeted therapy and BP onset varies considerably across published reports. In a systematic review of biologic-associated BP in patients with psoriasis, the mean latency was 5.12 ± 3.44 weeks for TNF-α inhibitors and 28.66 ± 26.27 weeks for ustekinumab [41]. Individual cases, however, range from onset shortly after a subsequent dose to development after several months of treatment. A compatible temporal relationship, improvement after drug withdrawal, and especially recurrence after re-exposure can strengthen a suspected drug association, but these features are inconsistently documented. Interpretation of dechallenge is further complicated when corticosteroids or other BP-directed therapies are started at the same time. Temporality should therefore be regarded as supportive evidence rather than as an independent criterion for establishing drug causality.

Most affected patients were male and developed BP in the sixth or seventh decade of life. Psoriasis usually preceded BP by 10–40 years, although synchronous onset and BP preceding erythrodermic psoriasis were occasionally reported. Typical manifestations included psoriatic plaques accompanied by tense bullae and erosions involving both lesional and uninvolved skin, with pruritus being a common symptom. Blister formation on pre-existing psoriatic plaques was reported in several cases, supporting the hypothesis that chronic psoriatic inflammation may predispose the basement membrane zone to subsequent autoantibody-mediated injury. Overall, clinical outcomes were favorable. Most patients achieved complete remission after withdrawal of the suspected agent, when appropriate, together with systemic corticosteroid therapy. Additional treatments, including methotrexate, dapsone, azathioprine, or tetracycline-class antibiotics, were required in some cases. Several patients were successfully switched to alternative targeted therapies without BP recurrence, highlighting the variable clinical effects of immune-targeted treatment in this setting. Importantly, established treatment evidence for these agents in psoriasis or BP individually should be distinguished from the predominantly case-based evidence currently available for concomitant psoriasis and BP.

### 4.1. IL-17 Inhibitors: Predominant Targeted Exposures in Concomitant Psoriasis and BP

IL-17A is a key effector cytokine in psoriasis, acting downstream of the IL-23/IL-17 axis to activate keratinocytes and other tissue-resident cells, thereby promoting the production of antimicrobial peptides, proinflammatory cytokines, and chemokines that sustain chronic skin inflammation [75]. Increasing evidence also supports a pathogenic role for IL-17A in BP. Chakievska et al. demonstrated elevated IL-17A expression in the serum and lesional skin of patients with BP, with Th17 cells identified as the major cellular source [27]. IL-17A promoted neutrophil activation and the release of proteolytic enzymes, including matrix metalloproteinase-9 (MMP-9) and neutrophil elastase, thereby facilitating dermoepidermal separation. In antibody-induced murine models, IL-17A blockade significantly reduced blister formation, further supporting its pathogenic role in BP. Together, these findings identify the IL-23/IL-17 axis as a shared pathogenic pathway linking psoriasis and BP and provide a biological rationale for IL-17-targeted therapy.

Clinical observations, however, suggest that the effects of IL-17 inhibition may vary across different immune contexts. IL-17 inhibitors have been reported in temporal association with BP onset or exacerbation in some patients. Although altered balance between Th17- and type 2 immune responses has been proposed as a possible explanation, this mechanism has not been directly demonstrated in the reported cases [76]. Among the 17 published cases involving IL-17 inhibitors included in this review, 7 were classified as temporal associations, 9 represented therapeutic use, and 1 reflected overlapping or confounded exposures. These data summarize published coexistence cases and should not be interpreted as estimates of BP risk or treatment efficacy associated with IL-17 inhibition. Overall, the divergent clinical effects of IL-17 blockade may reflect differences in the underlying immune environment, although this remains hypothetical.

### 4.2. IL-12/23 and IL-23 Inhibitors: Divergent Clinical Associations

IL-23 is a key upstream cytokine in psoriasis, promoting the differentiation, expansion, and maintenance of IL-17-producing T cells and thereby sustaining chronic keratinocyte-driven inflammation [1]. In BP, IL-23 appears to amplify inflammation primarily by regulating innate immune responses rather than directly inducing autoantibody production. Elevated IL-23 levels in serum and blister fluid have been associated with neutrophil activation and increased formation of NETs [28]. NETs accumulate at the dermal–epidermal junction and provide a rich source of proteolytic enzymes, including MMP-9 and neutrophil elastase. IL-23 also enhances IL-1β-associated inflammasome activation in monocytes and macrophages, further amplifying protease release and tissue injury [29]. Among the 13 published cases involving IL-12/23 or IL-23 inhibitors, 7 were classified as temporal associations, whereas 6 represented therapeutic use. These findings summarize published coexistence cases and should not be interpreted as estimates of risk or treatment efficacy associated with IL-23 inhibition. Similar to IL-17 blockade, the divergent clinical effects of IL-23 inhibition may reflect differences in the underlying immune environment. The mechanisms underlying these divergent clinical observations remain uncertain. Altered balance between Th17- and type 2 immune responses has been proposed as one possible explanation, but such a shift has not been directly demonstrated in the reported patients [77].

### 4.3. TNF-α Inhibitors: Early Observations and Paradoxical Presentations

TNF-α is a major proinflammatory cytokine in psoriasis, acting in concert with the IL-23/IL-17 axis to amplify and sustain cutaneous inflammation [1]. However, it is generally regarded as an inflammatory amplifier rather than a primary upstream driver of disease. In BP, TNF-α also contributes to leukocyte activation and tissue injury but does not appear to play a central pathogenic role, and current evidence does not support TNF-α as a primary therapeutic target [4]. Consequently, TNF-α inhibitors are used mainly for psoriasis and only occasionally in selected patients with BP. Among the three reported cases, BP developed after anti-TNF therapy in one patient and was considered possibly drug induced. In the other two cases, TNF-α inhibitors were introduced after BP diagnosis as part of combination or sequential treatment for concomitant psoriasis.

### 4.4. JAK Inhibitors: Emerging Small-Molecule Therapeutic Options

Four published case reports have described successful treatment with JAK inhibitors in patients with coexisting BP and psoriasis. In most cases, psoriasis preceded the onset of BP, whereas one patient developed recurrent psoriasis after BP. Conventional systemic therapies were limited by inadequate efficacy, intolerance, or contraindications. Treatment with baricitinib, tofacitinib, upadacitinib, or abrocitinib resulted in concurrent improvement of both BP and psoriasis. This therapeutic effect is biologically plausible. The Janus kinase/signal transducer and activator of transcription (JAK/STAT) pathway is a common downstream signaling pathway for multiple cytokines involved in both diseases, including IL-23-mediated Th17 responses in psoriasis and Th2-associated cytokine signaling in BP [78,79]. These case reports suggest potential therapeutic activity of JAK inhibitors in concomitant BP and psoriasis, but the evidence is insufficient to establish efficacy. JAK inhibitors differ in their selectivity and pharmacological profiles, and outcomes reported with individual agents cannot be generalized across the class. Safety is particularly important in older patients with BP, given the risks of serious infections, major adverse cardiovascular events, venous thromboembolism, and malignancy associated with JAK inhibition.

### 4.5. PDE4 Inhibitors: Potential Steroid-Sparing Small-Molecule Options

Two recent case reports have described successful treatment with the PDE4 inhibitor apremilast in patients with coexisting BP and psoriasis. In both cases, psoriasis preceded BP, and apremilast was used therapeutically. One patient relapsed shortly after treatment discontinuation but improved after reintroduction, whereas the other achieved sustained remission of both diseases, accompanied by reductions in anti-BP180 NC16A IgG and total immunoglobulin E (IgE) levels without recurrence during 36 weeks of follow-up [71,72]. Apremilast increases intracellular cyclic adenosine monophosphate and suppresses inflammatory cytokine production, thereby reducing keratinocyte-driven inflammation. This may decrease basement membrane antigen exposure and subsequent immune activation. In the latter case, reductions in anti-BP180 antibodies and total IgE, despite unchanged thymus and activation-regulated chemokine and eotaxin-1 levels and stable eosinophil counts, suggest that apremilast may act partly through modulation of humoral immunity rather than direct suppression of Th2-associated eosinophilic inflammation. Effects on neutrophil-driven inflammation may also contribute. However, with only two published cases, these observations remain hypothesis-generating and are insufficient to establish efficacy or a consistent mechanism. Apremilast may nonetheless represent a potential steroid-sparing option in selected patients.

### 4.6. Other Targeted Biologics

Published evidence for IL-36 receptor (IL-36R) blockade in patients with concomitant BP and psoriasis remains limited. The available evidence consists of a single case reported by Teshima et al., in which spesolimab was introduced after systemic corticosteroids and cyclosporine to treat generalized pustular psoriasis developing in a patient with long-standing psoriasis and BP, resulting in clinical remission [73]. This therapeutic strategy is biologically plausible. The IL-36/IL-36R axis is a key driver of neutrophilic inflammation in psoriasis, particularly generalized pustular psoriasis, through promotion of keratinocyte–myeloid cell interactions, IL-23/IL-17 signaling, and neutrophil recruitment. Selective IL-36R blockade may therefore suppress the dominant neutrophilic inflammatory response while having little direct effect on the autoantibody-mediated pathogenic mechanisms of BP [80].

Dupilumab selectively inhibits IL-4 receptor alpha (IL-4Rα) and downstream IL-4/IL-13 signaling, a central pathway in type 2 inflammation and the pathogenesis of BP. Although dupilumab has shown therapeutic benefit in BP more broadly, evidence specifically involving concomitant BP and psoriasis remains limited. Among the psoriasis–BP cases identified in this review, dupilumab was used therapeutically rather than reported as a suspected trigger. In the reported coexistence case, a patient with long-standing psoriasis and newly developed BP achieved partial remission of BP without worsening of psoriasis after dupilumab treatment [74]. This response is consistent with the prominent role of type 2 inflammation in BP, whereas psoriasis is predominantly driven by the IL-23/Th17 axis. Nevertheless, new-onset psoriasis, psoriasiform eruptions, and exacerbation of pre-existing psoriasis have been reported during dupilumab therapy as paradoxical cutaneous reactions. IL-4 can suppress Th17 responses and dendritic cell-derived IL-23 production, raising the possibility that IL-4Rα blockade may reduce this counter-regulatory effect and favor IL-23/Th17-associated inflammation in susceptible individuals [81]. The mechanisms underlying these reactions remain incompletely understood. The absence of psoriasis exacerbation in the reported coexistence case should therefore not be generalized, and psoriasis activity should be monitored during dupilumab treatment.

These observations support a pragmatic, disease-dominant treatment strategy for patients with concomitant BP and psoriasis. Therapy should primarily target the predominant inflammatory pathway while minimizing the risk of exacerbating the coexisting disease.

### 4.7. Current Controversies: Induction, Unmasking, or Coincidence?

Whether targeted therapies directly induce BP or instead unmask an underlying susceptibility remains uncertain. Temporal association alone cannot distinguish drug causation from acceleration of a pre-existing predisposition or coincidental disease onset. This uncertainty is underscored by reports in which targeting the same cytokine pathway has been associated with both BP onset or exacerbation and therapeutic benefit. These observations are therefore better interpreted as context-dependent associations rather than evidence of a consistent class effect. More robust causal inference will require longitudinal exposure data, reproducible temporal patterns, and, where available, informative dechalenge or rechallenge findings.

## 5. Therapeutic Considerations

Management of concomitant psoriasis and BP should be individualized according to the predominant disease, temporal sequence of onset, overall severity, age, comorbidities, previous treatment exposure, and the possibility of treatment-related disease activation. Each condition should primarily be managed according to established disease-specific guidelines, while evidence from concomitant psoriasis–BP cases should be considered supplementary rather than guideline-level. Current practice is informed mainly by case reports, small case series, observational studies, and extrapolation from disease-specific treatment strategies. Comparative evidence specifically addressing patients with both diseases remains scarce.

When BP predominates or newly develops, treatment should prioritize rapid control of blistering, pruritus, erosions, and secondary infection. High-potency topical corticosteroids are appropriate for localized disease, whereas more extensive BP may require systemic therapy [82]. Prolonged high-dose systemic corticosteroids should be minimized because of cumulative toxicity and the risk of psoriasis worsening during tapering or withdrawal [25]. Steroid-sparing therapy should be considered when longer-term control is required.

When psoriasis predominates and BP is mild or controlled, psoriasis treatment should be maintained while accounting for BP history [83]. If BP develops during targeted therapy, the diagnosis and temporal relationship should be reassessed before attributing causality. Discontinuation is reasonable when the association is convincing, particularly with improvement after withdrawal or recurrence after re-exposure. Otherwise, continuation or switching should be individualized according to the activity of both diseases, available alternatives, comorbidities, and monitoring capacity.

Psoriasis may also emerge during treatment of established BP, particularly after major changes in systemic corticosteroid exposure [25,82]. Newly introduced immunomodulatory therapies should be considered when timing is plausible, but temporal association alone is insufficient to establish causality. Abrupt withdrawal of BP therapy should generally be avoided unless a convincing drug–event relationship exists.

Switching therapeutic class may be considered when ongoing targeted treatment is needed and the previous agent is strongly suspected. Successful switching without BP recurrence has been reported [84], but such evidence remains limited to case reports. This is particularly relevant to IL-17- and IL-23-pathway inhibitors, for which both therapeutic benefit and BP onset or exacerbation have been described. Treatment choice should therefore be based on the individual disease course rather than an assumed class effect.

Conventional systemic therapies remain important when both diseases require treatment. Methotrexate is relevant because of its established efficacy in psoriasis and corticosteroid-sparing use in BP [85,86,87]. Cyclosporine or azathioprine may be considered when methotrexate is unsuitable, with careful attention to renal, hepatic, hematologic, infectious, and cumulative immunosuppressive risks [82,88].

Targeted and small-molecule therapies differ substantially in evidence base and regulatory status. Four published case reports have described improvement of both psoriasis and BP with individual JAK inhibitors [67,68,69,70]. However, these reports do not establish efficacy in BP, and their use specifically for BP remains off-label [89]. Apremilast has also been associated with concurrent improvement in individual reports [71,72], but evidence remains insufficient to establish efficacy in BP. IL-17- and IL-23-pathway inhibitors are established therapies for psoriasis but are not approved for BP. Dupilumab is approved for adult BP in the United States, although experience in patients with concomitant psoriasis remains limited [74].

Treatment decisions should be guided by the dominant clinical scenario rather than a fixed therapeutic hierarchy. Published case numbers should not determine treatment priority. When both diseases are active, treatment should be selected according to disease activity, approved indications, comorbidities, safety, and previous treatment response. After treatment initiation, withdrawal, or switching, activity of both BP and psoriasis should be monitored closely.

## 6. Limitations

This review has several limitations. Although a structured literature search was performed, the case synthesis is based largely on individual case reports and small case series and is therefore susceptible to publication and ascertainment bias. Unusual paradoxical reactions and successful therapeutic outcomes are more likely to be reported than uneventful treatment exposures. Important clinical variables, including concomitant therapies, disease severity, prior phototherapy, comorbidities, diagnostic evaluation, autoantibody testing, and follow-up duration, were inconsistently reported. The exposure categories used in this review— “Temporal association”, “Therapeutic use”, and “Overlapping exposure”—should therefore be regarded as descriptive rather than causal classifications. Causal grading of temporally associated cases was also limited by incomplete information on latency, dechallenge, rechallenge, concomitant medications, and alternative causes. The available case literature cannot reliably estimate incidence, relative risk, comparative safety, treatment efficacy, or causality for individual agents or therapeutic classes. Finally, restriction to English-language publications may have introduced language bias and led to omission of relevant reports.

## 7. Future Directions

Future research should prioritize prospective multicenter and longitudinal studies with standardized diagnostic criteria, disease-severity measures, and detailed treatment histories. Studies of patients receiving biologic or small-molecule therapies should include defined exposure denominators, appropriate comparators, and systematic assessment of latency, withdrawal, switching, and recurrence to better distinguish treatment-related BP from background susceptibility and surveillance bias.

Longitudinal biomarker and mechanistic studies are also needed to determine whether immunological and BMZ changes precede BP onset. Serial analysis of serological, cellular, and tissue markers, including changes in BMZ architecture and autoantigen accessibility, may help identify early events in BP development. Such studies could clarify whether BMZ remodeling and epitope spreading contribute causally to psoriasis-associated BP or simply reflect shared inflammatory susceptibility.

## 8. Conclusions

The coexistence of psoriasis and BP is clinically important and immunologically complex and is unlikely to be explained by chance alone. Current evidence supports a bidirectional epidemiological association between psoriasis and BP, whereas genetic data suggest an asymmetric susceptibility. Chronic inflammation, immune plasticity, BMZ alterations, and therapeutic exposures have been proposed as potential contributors, but their causal roles remain uncertain. Targeted therapies have expanded treatment options while also revealing paradoxical immune responses in some patients. IL-17-, IL-23-, and TNF-α-targeted therapies have been associated with both therapeutic benefit and BP onset or exacerbation in published reports, and the basis for these contrasting outcomes remains unclear. One possibility is that treatment effects vary according to the prevailing immune milieu, although this has not been mechanistically established. Small-molecule therapies, particularly JAK and PDE4 inhibitors, have shown corticosteroid-sparing potential in isolated reports, but the overall evidence remains limited to case reports and small observational studies and is insufficient to support firm treatment recommendations. Future studies should define the mechanisms underlying immune plasticity and paradoxical inflammation to improve risk stratification, guide individualized treatment, and support the safer use of biologic and targeted therapies in patients with concomitant psoriasis and BP.

## Figures and Tables

**Figure 1:**
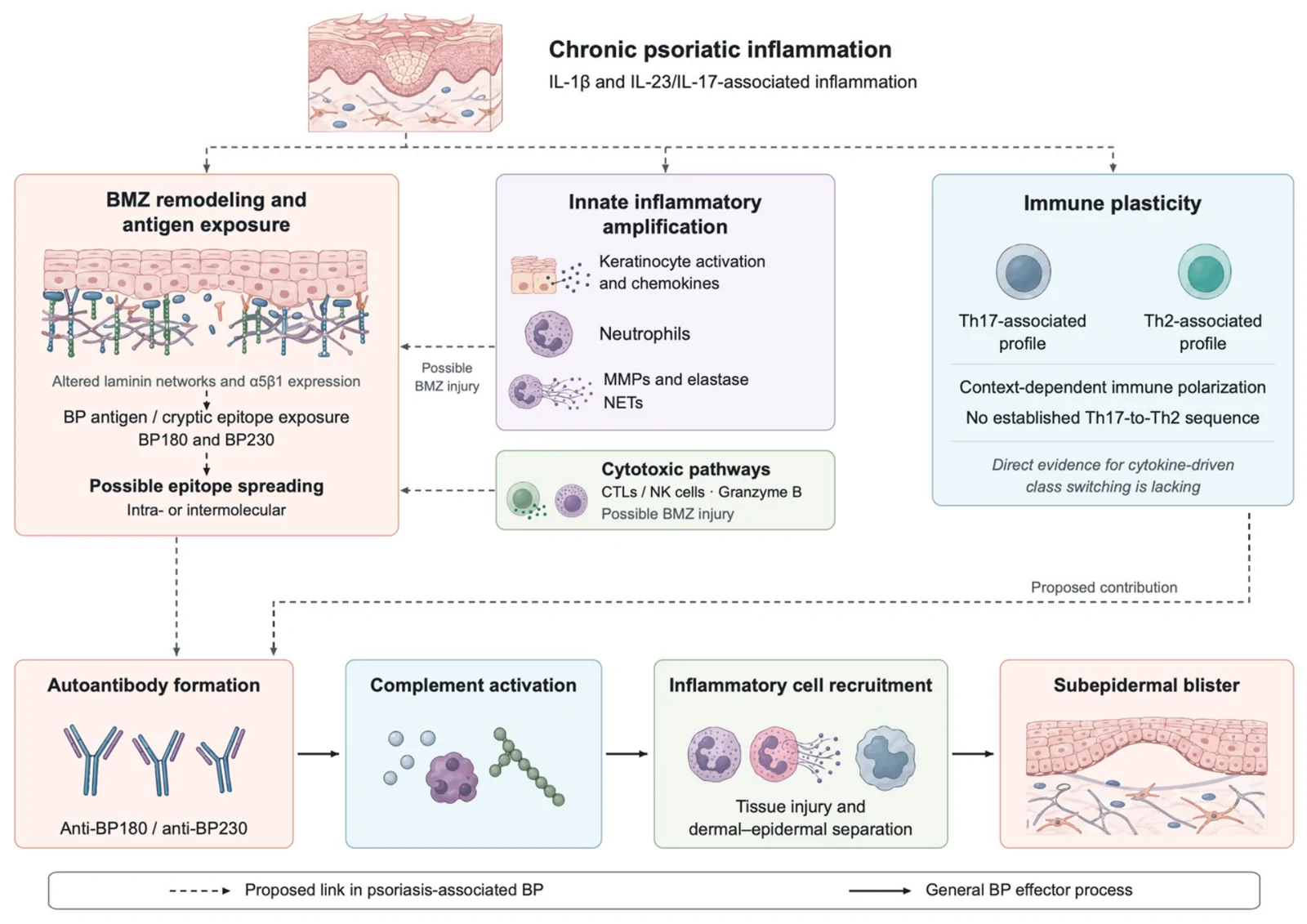
Proposed immunopathogenic framework linking psoriasis and bullous pemphigoid (BP). The sequence illustrated represents a biologically plausible, non-linear model and should not be interpreted as a demonstrated temporal progression in individual patients. Chronic psoriatic inflammation may contribute to basement membrane zone (BMZ) remodeling, potentially increasing the accessibility of BP-associated autoantigens and facilitating epitope spreading. Autoantibody formation, complement activation, inflammatory-cell recruitment, and protease release may subsequently contribute to dermal–epidermal separation and blister formation. IL-17/IL-23 signaling, neutrophil extracellular traps, type 2 inflammation, and cytotoxic mediators may further amplify these processes. Abbreviations: BP180, bullous pemphigoid antigen 180; BP230, bullous pemphigoid antigen 230; CTLs, cytotoxic T lymphocytes; IL, interleukin; LAD-1, linear IgA disease antigen 1; MMPs, matrix metalloproteinases; NK, natural killer; Th, T helper. This figure was created by the authors using Adobe Illustrator 2025 (version 29.0; Adobe Inc., San Jose, CA, USA).

**Table 1:** Principal epidemiological studies examining the association between psoriasis and BP.

Study	Region	Study Design	Sample Size	Main Finding	Major Limitations
Grattan, 1985 [5]	UK	Retrospective matched case–control study	62 pemphigoids; 62 controls	Psoriasis: 11% vs. 0%; *p* < 0.01	Small sample; historical diagnostic framework; limited confounder adjustment
Phan et al., 2019 [9]	Multiple regions	Systematic review and meta-analysis of 4 case–control studies	4035 BP; 19,215 controls	OR 2.5, 95% CI 1.4–4.6	Small number of included studies; heterogeneity among observational studies
Ho et al., 2019 [7]	Taiwan	Nationwide retrospective cohort	109,777 psoriasis; 109,777 controls	HR 3.05, 95% CI 2.10–4.43	Claims-based diagnoses; residual confounding; incomplete clinical and treatment detail
Kridin et al., 2020 [10]	Israel	Population-based retrospective cohort and case–control analyses	3924 BP; 19,280 controls	BP→psoriasis: HR 2.60, 95% CI 1.59–4.27; psoriasis→BP: adjusted OR 1.53, 95% CI 1.14–2.06	Retrospective routinely collected data; limited disease-severity and treatment information
Kim et al., 2025 [11]	South Korea	Nationwide population-based case–control study	5066 BP; 10,132 controls	Psoriasis before BP: OR 3.15, 95% CI 2.58–3.85	Administrative-code ascertainment; retrospective design; potential residual confounding

Abbreviations: BP, bullous pemphigoid; CI, confidence interval; HR, hazard ratio; OR, odds ratio. Limitations were summarized according to study design and the information available in the original publications.

**Table 2:** Reported clinical and immunopathological characteristics of bullous pemphigoid associated with psoriasis.

Characteristic	Reported Findings in Psoriasis-Associated BP	Evidence
Age at BP onset	Earlier onset reported in two cohorts (74.4 vs. 76.8; 68.5 vs. 78.0 years)	Population-based study [10]; hospital-based case–control study [14]
Sex distribution	Higher male proportion reported (53.5% vs. 42.2%)	Population-based study [10]
Disease chronology	Psoriasis usually precedes BP; mean latency varied from 2.86 to 25.2 years across cohorts	Cohort studies [7,10,14]
Mucosal involvement	Numerically higher frequency (33.3% vs. 15.4%), but not statistically significant	Hospital-based case–control study [14]
Treatment burden	Greater long-term systemic and topical corticosteroid use reported	Population-based study [10]
Immunopathology	Atypical DIF findings, including linear C3 deposition without detectable IgG	Case reports/small series [16]
Prior treatment exposure	Phototherapy, conventional systemic agents, and biologics reported before BP onset	Cohort and case reports [7,17,18]

Abbreviations: BMZ, basement membrane zone; BP, bullous pemphigoid; BP180, bullous pemphigoid antigen 180; BP230, bullous pemphigoid antigen 230; C3, complement component 3; DIF, direct immunofluorescence; IgG, immunoglobulin G. Reported findings are derived from heterogeneous observational studies and case reports and should be interpreted as clinical tendencies rather than defining features of psoriasis-associated BP.

**Table 3:** Summary and evidence basis of published patient-level reports involving targeted therapy exposure in patients with concomitant psoriasis and BP.

Targeted Therapy Class	No. of Cases	Descriptive Exposure Category	Evidence Basis	References	Key Interpretive Points
IL-17 inhibitors	17	Temporal association: 7; Therapeutic use: 9; Overlapping exposure: 1;	Case reports and small case series	[42,43,44,45,46,47,48,49,50,51,52,53,54,55]	Most frequently reported class; both temporally associated BP onset/exacerbation and successful disease control have been described.
IL-12/23 or IL-23 inhibitors	13	Temporal association: 7; Therapeutic use: 6;	Case reports and small multi-patient reports	[18,56,57,58,59,60,61,62,63,64,65]	Reported in both temporal association and therapeutic contexts; the divergent observations remain hypothesis-generating.
TNF-α inhibitors	3	Temporal association: 1; Therapeutic use: 1; Overlapping exposure: 1;	Case reports	[17,34,66]	Evidence is limited and partly confounded by concomitant treatments and prior disease history.
JAK inhibitors	4	Therapeutic use: 4;	Case reports	[67,68,69,70]	Reported cases describe concurrent improvement of psoriasis and BP, but efficacy remains unestablished.
PDE4 inhibitors	2	Therapeutic use: 2;	Case reports	[71,72]	Two case reports provide hypothesis-generating evidence for a potential steroid-sparing role.
Other targeted agents	2	Therapeutic use: 2;	Case reports	[73,74]	Includes IL-36R blockade and IL-4Rα blockade in selected clinical contexts.
Total	41	Temporal association: 15; Therapeutic use: 24; Overlapping exposure: 2;	Predominantly case reports and small case series	—	Counts reflect published-case distribution and should not be interpreted as estimates of true drug-specific risk, safety, or efficacy.

Abbreviations: BP, bullous pemphigoid; IL, interleukin; JAK, Janus kinase; PDE4, phosphodiesterase 4; TNF-α, tumor necrosis factor alpha. Evidence basis refers to the 42 patient-level cases included in this synthesis and pertains specifically to concomitant psoriasis and BP; it should not be extrapolated to established treatment evidence for either disease individually. Pharmacovigilance analyses and population-level studies without extractable patient-level data were excluded from the case counts. ‘Temporal association’ indicates that targeted therapy preceded new-onset or worsening disease with a plausible causal relationship in the original report; ‘therapeutic use’ indicates administration for disease control; and ‘overlapping exposure’ indicates an unclear temporal or mechanistic relationship to disease onset or exacerbation. These categories are descriptive and do not establish causality. Published case counts lack exposure denominators and should not be interpreted as estimates of incidence, relative risk, comparative safety, or efficacy.

## Data Availability

No new primary data were generated. Patient-level data extracted from published reports were classified and synthesized, and the derived dataset is provided in the Supplementary Materials.

## Supplementary Materials

The following supporting information can be downloaded at: https://www.mdpi.com/article/10.3390/biom16101391/s1, Figure S1: Literature search framework and derivation of the patient-level targeted-therapy dataset; Table S1: Complete Search Strategies; Table S2: Published Cases of Concomitant Psoriasis and Bullous Pemphigoid Associated with Targeted Therapy Exposure.

## Abbreviations

The following abbreviations are used in this manuscript:

BMZ	basement membrane zone
BP	bullous pemphigoid
BP180	bullous pemphigoid antigen 180
BP230	bullous pemphigoid antigen 230
C3	complement component 3
CI	confidence interval
CR	complete remission
DIF	direct immunofluorescence
GPP	generalized pustular psoriasis
HLA	human leukocyte antigen
IgE	immunoglobulin E
IgG	immunoglobulin G
IL	interleukin
IL-4Rα	interleukin-4 receptor alpha
IL-36R	interleukin-36 receptor
JAK	Janus kinase
JAK/STAT	Janus kinase/signal transducer and activator of transcription
LAD-1	linear IgA disease antigen 1
MMP-9	matrix metalloproteinase-9
NC16A	non-collagenous 16A domain
NETs	neutrophil extracellular traps
OR	odds ratio
PDE4	phosphodiesterase 4
PR	partial remission
Th	T helper
TNF-α	tumor necrosis factor alpha
Treg	regulatory T cell

## References

[1] Armstrong A.W., Blauvelt A., Callis Duffin K., Huang Y.H., Savage L.J., Guo L., Merola J.F. (2025). Psoriasis. Nat. Rev. Dis. Primers.

[2] Furue K., Ito T., Tsuji G., Kadono T., Nakahara T., Furue M. (2018). Autoimmunity and autoimmune co-morbidities in psoriasis. Immunology.

[3] Werth V.P., Murrell D.F., Joly P., Ardeleanu M., Hultsch V. (2024). Bullous pemphigoid burden of disease, management and unmet therapeutic needs. J. Eur. Acad. Dermatol. Venereol..

[4] Akbarialiabad H., Schmidt E., Patsatsi A., Lim Y.L., Mosam A., Tasanen K., Yamagami J., Daneshpazhooh M., De D., Cardones A.R.G. (2025). Bullous pemphigoid. Nat. Rev. Dis. Primers.

[5] Grattan C.E. (1985). Evidence of an association between bullous pemphigoid and psoriasis. Br. J. Dermatol..

[6] Ohata C., Ishii N., Koga H., Fukuda S., Tateishi C., Tsuruta D., Furumura M., Hashimoto T. (2015). Coexistence of autoimmune bullous diseases (AIBDs) and psoriasis: A series of 145 cases. J. Am. Acad. Dermatol..

[7] Ho Y.H., Hu H.Y., Chang Y.T., Li C.P., Wu C.Y. (2019). Psoriasis is associated with increased risk of bullous pemphigoid: A nationwide population-based cohort study in Taiwan. J. Dermatol..

[8] Maronese C.A., Cassano N., Genovese G., Foti C., Vena G.A., Marzano A.V. (2023). The Intriguing Links between Psoriasis and Bullous Pemphigoid. J. Clin. Med..

[9] Phan K., Goyal S., Murrell D.F. (2019). Association between bullous pemphigoid and psoriasis: Systematic review and meta-analysis of case-control studies. Australas. J. Dermatol..

[10] Kridin K., Ludwig R.J., Schonmann Y., Damiani G., Cohen A.D. (2020). The Bidirectional Association Between Bullous Pemphigoid and Psoriasis: A Population-Based Cohort Study. Front. Med..

[11] Kim B.R., Lee K.H., Paik K., Kim M., Bae J.M., Choi C.W., Youn S.W. (2025). Comorbid diseases in bullous pemphigoid: A population-based case-control study. J. Dermatol..

[12] Wang X., Zhu Z. (2025). Causal Relationship Between Psoriasis and Bullous Pemphigoid: A Mendelian Randomization Analysis. Dermatol. Pract. Concept..

[13] Zhang A., Yang Z., Huang T., Wang M. (2024). Causal association between psoriasis vulgaris and bullous pemphigoid: A two-sample bidirectional Mendelian randomization study. Front. Immunol..

[14] Kridin K., Bergman R. (2017). Association between bullous pemphigoid and psoriasis: A case-control study. J. Am. Acad. Dermatol..

[15] Niebel D., Wilsmann-Theis D., Bieber T., Berneburg M., Wenzel J., Braegelmann C. (2022). Bullous Pemphigoid in Patients Receiving Immune-Checkpoint Inhibitors and Psoriatic Patients-Focus on Clinical and Histopathological Variation. Dermatopathology.

[16] Im D., Ueda K., Niwa H., Tanaka K., Iwata H. (2025). A case of bullous pemphigoid associated with psoriasis showing no tissue-bound immunoglobulin G on the inflamed skin. J. Dermatol..

[17] Gambichler T., Noldes K., Boms S. (2024). Bullous pemphigoid and lichen planus pemphigoides in monozygotic twins with a long-standing history of psoriasis. Clin. Exp. Dermatol..

[18] Querol-Cisneros E., Moreno-Artero E., Rodríguez-Garijo N., Tomás-Velázquez A., Querol I., Ishii N., Hashimoto T., España A. (2021). Bullous pemphigoid without detection of autoantibodies in a patient with psoriasis under ustekinumab. J. Dtsch. Dermatol. Ges..

[19] Kridin K., Ahmed A.R. (2019). Anti-p200 Pemphigoid: A Systematic Review. Front. Immunol..

[20] Goletz S., Pigors M., Lari T.R., Hammers C.M., Wang Y., Emtenani S., Aumailley M., Holtsche M.M., Stang F.H., Weyers I. (2024). Laminin β4 is a constituent of the cutaneous basement membrane zone and additional autoantigen of anti-p200 pemphigoid. J. Am. Acad. Dermatol..

[21] Borradori L., Van Beek N., Feliciani C., Tedbirt B., Antiga E., Bergman R., Böckle B.C., Caproni M., Caux F., Chandran N.S. (2022). Updated S2 K guidelines for the management of bullous pemphigoid initiated by the European Academy of Dermatology and Venereology (EADV). J. Eur. Acad. Dermatol. Venereol..

[22] Solimani F., Pollmann R., Ishii N., Eming R., Hashimoto T., Schmidt T., Hertl M. (2019). Diagnosis of anti-laminin γ-1 pemphigoid by immunoblot analysis. J. Eur. Acad. Dermatol. Venereol..

[23] Timofte M.A., Căruntu C., Bălăceanu-Gurău B., Mărgăritescu I., Giurcăneanu C., Mihai M.M. (2025). Bullous Pemphigoid Overlapping Psoriasis Vulgaris: A Rare Case Report and Literature Review. Clin. Pract..

[24] Orzan O.A., Tutunaru C.V., Ianoși S.L. (2025). Understanding the Intricate Pathophysiology of Psoriasis and Related Skin Disorders. Int. J. Mol. Sci..

[25] Yasukawa S., Dainichi T., Kokuba H., Moroi Y., Urabe K., Hashimoto T., Furue M. (2009). Bullous pemphigoid followed by pustular psoriasis showing Th1, Th2, Treg and Th17 immunological changes. Eur. J. Dermatol..

[26] Liu Y., Wang H., Taylor M., Cook C., Martínez-Berdeja A., North J.P., Harirchian P., Hailer A.A., Zhao Z., Ghadially R. (2022). Classification of human chronic inflammatory skin disease based on single-cell immune profiling. Sci. Immunol..

[27] Chakievska L., Holtsche M.M., Künstner A., Goletz S., Petersen B.S., Thaci D., Ibrahim S.M., Ludwig R.J., Franke A., Sadik C.D. (2019). IL-17A is functionally relevant and a potential therapeutic target in bullous pemphigoid. J. Autoimmun..

[28] Giusti D., Bini E., Terryn C., Didier K., Le Jan S., Gatouillat G., Durlach A., Nesmond S., Muller C., Bernard P. (2019). NET Formation in Bullous Pemphigoid Patients With Relapse Is Modulated by IL-17 and IL-23 Interplay. Front. Immunol..

[29] Le Jan S., Muller C., Plee J., Durlach A., Bernard P., Antonicelli F. (2019). IL-23/IL-17 Axis Activates IL-1β-Associated Inflammasome in Macrophages and Generates an Auto-Inflammatory Response in a Subgroup of Patients With Bullous Pemphigoid. Front. Immunol..

[30] Muramatsu K., Ujiie H., Kobayashi I., Nishie W., Izumi K., Ito T., Yoshimoto N., Natsuga K., Iwata H., Shimizu H. (2018). Regulatory T-cell dysfunction induces autoantibodies to bullous pemphigoid antigens in mice and human subjects. J. Allergy Clin. Immunol..

[31] Haeberle S., Wei X., Bieber K., Goletz S., Ludwig R.J., Schmidt E., Enk A.H., Hadaschik E.N. (2018). Regulatory T-cell deficiency leads to pathogenic bullous pemphigoid antigen 230 autoantibody and autoimmune bullous disease. J. Allergy Clin. Immunol..

[32] Caca-Biljanovska N., Arsovska-Bezhoska I., V’Lckova-Laskoska M. (2016). PUVA-induced Bullous Pemphigoid in Psoriasis. Acta Dermatovenerol. Croat..

[33] Ceryn J., Skibińska M., Barasiñska P., Noweta M., Narbutt J., Lesiak A. (2022). UVB-induced bullous pemphigoid in a patient with psoriasis. Postep. Dermatol. Alergol..

[34] Saraceno R., Citarella L., Spallone G., Chimenti S. (2008). A biological approach in a patient with psoriasis and bullous pemphigoid associated with losartan therapy. Clin. Exp. Dermatol..

[35] Chen W., Peng C., Zheng J., Lu X., Ding Y., Su L. (2022). Bullous Pemphigoid Associated with Erythrodermic Psoriasis: A Case Report. Clin. Cosmet. Investig. Dermatol..

[36] Martora F., Battista T., Fabbrocini G., Megna M. (2023). Concomitant Severe Psoriasis and Bullous Pemphigoid Induced by COVID-19. Trop. Med. Infect. Dis..

[37] Okahashi K., Oiso N., Ishii N., Uchida S., Matsuda H., Hashimoto T., Kawada A. (2015). Bullous pemphigoid associated with psoriasis: A possible example of an inverse intramolecular epitope-spreading phenomenon. J. Dermatol..

[38] Inamura E., Tsujiwaki M., Ujiie H., Nishie W., Hata H., Shimizu H., Iwata H. (2021). Bullous pemphigoid associated with psoriasis showing marked neutrophilic infiltrates. J. Dtsch. Dermatol. Ges..

[39] Dainichi T., Kabashima K. (2018). Interaction of Psoriasis and Bullous Diseases. Front. Med..

[40] Gleave A., Granville D.J. (2023). Granzyme B in Autoimmune Skin Disease. Biomolecules.

[41] Husein-ElAhmed H., Steinhoff M. (2022). Bullous pemphigoid induced by biologic drugs in psoriasis: A systematic review. J. Dermatol. Treat..

[42] Paschou I.A., Drantaki V.I., Gerochristou M., Stefanaki I., Lingria A., Stratigos A.J., Nikolaou V. (2025). Dual Immune-Related Toxicities: Secukinumab-Induced Bullous Pemphigoid Following ICI-Associated Psoriasis in a Cancer Patient. Int. J. Dermatol..

[43] Okamura K., Saito T., Arai Y., Suto H., Suzuki T. (2025). Effective management of coexisting bullous pemphigoid and psoriasis with bimekizumab. J. Dermatol..

[44] Cao S., Yang B., Liu Y., Liu H., Zhang F. (2025). Combined biological therapy with dupilumab and secukinumab for coexisting bullous pemphigoid and psoriasis. Indian. J. Dermatol. Venereol. Leprol..

[45] Lugli A.P., Caldarola G., Falco G.M., Montedoro C., Mulas C., De Simone C. (2024). Anti-IL-17 monoclonal antibodies and bullous pemphigoid: Treatment or causal agents? A case series and review of the literature. Drugs Context.

[46] Wang G., Hu X., Han S., Zhou M. (2024). A case report of bullous pemphigoid following secukinumab therapy for a patient with psoriasis. J. Dermatol. Treat..

[47] Wang R., Wang A., Wang M. (2023). Two cases with bullous pemphigoid relapsed after using interleukin-17A inhibitors for psoriasis: A paradoxical reaction. J. Dermatol..

[48] Kochi Y., Miyachi H., Tagashira R., Koga H., Ishii N., Sugiura K., Ikeda J.I., Matsue H., Inozume T. (2023). Simultaneous development of generalized pustular psoriasis and pemphigoid with multiple autoantibodies in a complete responder of pembrolizumab for lung cancer. J. Dermatol..

[49] Xiao Y., Gu Y., Xia D., Zhou X., Li W. (2023). Ixekizumab successfully treated refractory psoriasis concurrent bullous pemphigoid. J. Dermatol..

[50] Drenovska K., Valeva E., Shahid M., Vassileva S. (2023). Case Report: Coexistence of bullous pemphigoid and psoriasis: Therapeutic challenge and IL17A-targeted parallel treatment strategy. Front. Med..

[51] Yun J.S., Scardamaglia L., Tan C.G., McCormack C.J. (2022). Successful secukinumab treatment of active bullous pemphigoid and chronic severe psoriasis: A case report. Australas. J. Dermatol..

[52] Lu L., Yu Y., Zhang J., Fan X., Qi Y., Lin B. (2022). Incidental amelioration of bullous pemphigoid during ixekizumab treatment for psoriasis. J. Dermatol..

[53] Otani Y., Hayashi S., Kaneko Y., Koike M., Gommori T., Hamasaki Y., Igawa K. (2021). Pustular psoriasis associated with bullous pemphigoid with fluctuating anti-BP180 antibody titre according to the condition of pustular lesions. Eur. J. Dermatol..

[54] Kamata M., Asano Y., Shida R., Maeda N., Yoshizaki A., Miyagaki T., Kawashima T., Tada Y., Sato S. (2019). Secukinumab decreased circulating anti-BP180-NC16a autoantibodies in a patient with coexisting psoriasis vulgaris and bullous pemphigoid. J. Dermatol..

[55] Ho P.H., Tsai T.F. (2017). Development of bullous pemphigoid during secukinumab treatment for psoriasis. J. Dermatol..

[56] Xi R., Cao Y., Zhuang Q., Ma L., Tao M., Li Y. (2025). Bullous pemphigoid induced by anti-IL-23 monoclonal antibody in a psoriatic patient: A case report. Front. Med..

[57] Sugai S., Takamura S., Fukuda T. (2025). Effect of IL-23p19 Inhibitors for Psoriasis With Bullous Pemphigoid: Clinical Insights From Two Cases. J. Dermatol..

[58] Rodríguez-Cuadrado F.J., Roustan-Gullón G., Suárez-Massa D., Hospital-Gil M. (2024). Multirefractory bullous pemphigoid, psoriasis and psoriatic arthritis successfully treated with guselkumab. An. Bras. Dermatol..

[59] Cole C., Amber K.T. (2023). Two patients with concomitant bullous pemphigoid and psoriasis successfully treated by IL-23 inhibition. J. Eur. Acad. Dermatol. Venereol..

[60] Kong L., Huang D., Lu J., Zhang Y., Li Y., Yi X., Shi Y. (2023). Development of bullous pemphigoid during treatment of psoriasis with ustekinumab: A case report and literature review. Front. Med..

[61] Fukada N., Suzuki T., Aoyama K., Kinjo A., Yamazaki K., Sato T. (2023). Bullous pemphigoid during the treatment of psoriasis vulgaris with risankizumab. J. Dermatol..

[62] Burlando M., Capurro N., Herzum A., Cozzani E., Parodi A. (2022). Guselkumab-associated bullous pemphigoid in a psoriasis patient: A case report and review of the literature. Dermatol. Ther..

[63] Marin M., Alzueta N., Castresana M., Gascón A., Pío M. (2021). Bullous pemphigoid induced by ustekinumab: A case report. Eur. J. Hosp. Pharm..

[64] Loget J., Plée J., Antonicelli F., Bernard P. (2017). A successful treatment with ustekinumab in a case of relapsing bullous pemphigoid associated with psoriasis. J. Eur. Acad. Dermatol. Venereol..

[65] Nakayama C., Fujita Y., Watanabe M., Shimizu H. (2015). Development of bullous pemphigoid during treatment of psoriatic onycho-pachydermo periostitis with ustekinumab. J. Dermatol..

[66] Tirado-Sánchez A., Bonifaz A. (2020). Simultaneous Bullous Pemphigoid and Vitiligo Associated with Adalimumab Therapy in a Patient with Psoriasis Vulgaris. Indian. Dermatol. Online J..

[67] Hu Y.Q., Zhang J.Z., Zhao Y. (2026). Successful Treatment of Concurrent Bullous Pemphigoid and Psoriasis Vulgaris with Abrocitinib: A Case Report and Literature Review. Clin. Cosmet. Investig. Dermatol..

[68] Su F., Wang T., Qin Q., Xie Z. (2024). Upadacitinib for the management of bullous pemphigoid coexisting with psoriasis vulgaris: A case report and literature review. J. Dermatol. Treat..

[69] Li H., Wang H., Qiao G., Liu Y., Zhang F., Pan F. (2023). Concurrent bullous pemphigoid and psoriasis vulgaris successfully treated with Janus kinase inhibitor tofacitinib: A case report and review of the literature. Int. Immunopharmacol..

[70] Xiao Y., Xiang H., Li W. (2022). Concurrent bullous pemphigoid and plaque psoriasis successfully treated with Janus kinase inhibitor Baricitinib. Dermatol. Ther..

[71] Kumagai H., Ototake Y., Watanabe T., Takamura N., Inoue M., Shimada M., Yamaguchi Y. (2026). A Case of Bullous Pemphigoid Coexisted With Psoriasis Vulgaris Successfully Treated by Apremilast. J. Dermatol..

[72] Kitayama S., Makino T., Takemoto K., Mizawa M., Shirasaki F., Shimizu T. (2026). Apremilast Associated Clinical Improvement in Bullous Pemphigoid With Plaque Psoriasis. J. Dermatol..

[73] Teshima R., Saito-Sasaki N., Hitaka T., Sawada Y. (2024). Spesolimab in the Management of Generalized Pustular Psoriasis With Concurrent Bullous Pemphigoid and Psoriasis. Cureus.

[74] Liu J.H., Gao Q., Ma W.Y., Cheng Z.L., Luo N.N., Hao P.S. (2023). Successful Treatment of Psoriasis Combined with Bullous Pemphigoid with Dupilumab: A Case Report. Clin. Cosmet. Investig. Dermatol..

[75] Blauvelt A., Chiricozzi A. (2018). The Immunologic Role of IL-17 in Psoriasis and Psoriatic Arthritis Pathogenesis. Clin. Rev. Allergy Immunol..

[76] Heidari N., Hosseini S., Heidari A., Ghane Y., Daneshpazhooh M., Mahmoudi H. (2025). A systematic review of the role of interleukin-17 inhibitors in bullous pemphigoid: Therapeutic and paradoxical effects. Arch. Dermatol. Res..

[77] Maronese C.A., Valenti M., Moltrasio C., Romagnuolo M., Ferrucci S.M., Gilliet M., Costanzo A., Marzano A.V. (2024). Paradoxical Psoriasis: An Updated Review of Clinical Features, Pathogenesis, and Treatment Options. J. Invest. Dermatol..

[78] Lin X., Li X., Zhai Z., Zhang M. (2025). JAK-STAT pathway, type I/II cytokines, and new potential therapeutic strategy for autoimmune bullous diseases: Update on pemphigus vulgaris and bullous pemphigoid. Front. Immunol..

[79] Nogueira M., Puig L., Torres T. (2020). JAK Inhibitors for Treatment of Psoriasis: Focus on Selective TYK2 Inhibitors. Drugs.

[80] Ahmad F., Alam M.A., Ansari A.W., Jochebeth A., Leo R., Al-Abdulla M.N., Al-Khawaga S., AlHammadi A., Al-Malki A., Al Naama K. (2024). Emerging Role of the IL-36/IL-36R Axis in Multiple Inflammatory Skin Diseases. J. Invest. Dermatol..

[81] Hahn M., Ghoreschi K. (2017). The role of IL-4 in psoriasis. Expert. Rev. Clin. Immunol..

[82] Hsieh C.Y., Tsai T.F. (2022). Management of Coexisting Bullous Pemphigoid and Psoriasis: A Review. Am. J. Clin. Dermatol..

[83] Nast A., Altenburg A., Augustin M., Bachmann F., Boehncke W.H., Cornberg M., Geerdes-Fenge H., Häcker B., Härle P., Klaus J. (2026). S3 Guideline for the treatment of psoriasis vulgaris, adapted from EuroGuiDerm—Part 1: Treatment recommendations and monitoring. J. Dtsch. Dermatol. Ges..

[84] Le Guern A., Alkeraye S., Vermersch-Langlin A., Coupe P., Vonarx M. (2015). Bullous pemphigoid during ustekinumab therapy. JAAD Case Rep..

[85] Ma W., Li C., Miao H. (2026). Glucocorticoids Combined with Methotrexate in the Treatment of Psoriasis Merged with Bullous Pemphigoid: A Case Report and Literature Review. Clin. Case Rep..

[86] Iskandarli M., Gerceker Turk B., Yaman B., Ozturk G. (2015). Pemphigoid Diseases as a Sign of Active Psoriasis: A Case Report and Brief Review. Dermatology.

[87] Vargas P., Giacaman P., Fernández J., Morales C. (2019). Bullous pemphigoid associated with psoriasis: A good response to methotrexate. An. Bras. Dermatol..

[88] Primka E.J., Camisa C. (1998). Psoriasis and bullous pemphigoid treated with azathioprine. J. Am. Acad. Dermatol..

[89] Lovegrove F.E., Sauder M.B., Mufti A., Beecker J., Dutz J.P., Kirchhof M.G., Mydlarski P.R., Purdy K.S., Lansang P. (2025). Navigating Bullous Pemphigoid: Consensus Recommendations for Diagnosis and Management-A Canadian Perspective. J. Cutan. Med. Surg..

