# Supplementary material for: Psoriasis and Bullous Pemphigoid: An Updated Review of Clinical Association, Targeted Therapy Exposures and Therapeutic Implications
Source: Biomolecules. 2026 Sep 24;16(10):1391. doi: 10.3390/biom16101391 (PMC13702189; doi:10.3390/biom16101391)
Supplement: Supplementary file 1 [file biomolecules-16-01391-s001.zip › biomolecules-4489626-supplementary Figure S1.pdf]

Figure S1. Literature search framework and derivation of the patient-level targeted-therapy dataset

A. Literature search framework

Epidemiological evidence

PubMed/MEDLINE: n = 120

Web of Science: n = 166

Google Scholar: first 200 relevance-ranked records screened

Mechanistic evidence

PubMed/MEDLINE: n = 262

Web of Science: n = 295

Google Scholar: first 200 relevance-ranked records screened

Patient-level targeted-therapy search

PubMed/MEDLINE: n = 459

Web of Science: n = 463

Google Scholar: first 200 relevance-ranked records screened

Reference-list checking: no additional unique records

B. Derivation of the 41-patient targeted-therapy dataset

Records identified from targeted-therapy case search
PubMed/MEDLINE: 459
Web of Science: 463
Google Scholar: 200

Total: n = 1,122

↓

Exact duplicate records removed
DOI, PMID, or normalized title
n = 449

↓

Unique records after exact deduplication
n = 673

↓

Eligibility assessment for patient-level synthesis

Inclusion criteria

- psoriasis + confirmed BP
- targeted-therapy exposure
- extractable patient-level information
- sufficient detail on chronology, treatment exposure, clinical course, or outcome

Principal exclusion criteria

- no concomitant psoriasis and BP
- no eligible targeted-therapy exposure
- insufficient patient-level information
- other autoimmune blistering disease without confirmed BP
- duplicate/overlapping patient report
- publication after 14 April 2026 or unavailable English-language full text

↓

Included publications
n = 35

↓

Individual patients included
n = 41

↓

Distribution by targeted-therapy class

- IL-17 inhibitors: 17
- IL-12/23 or IL-23 inhibitors: 13
- JAK inhibitors: 4
- TNF-α inhibitors: 3
- PDE4 inhibitors: 2
- Other targeted agents: 2

Descriptive exposure category

- Presumed trigger: 15
- Therapeutic use: 24
- Overlapping exposure: 2
