# Supplementary material for: Psoriasis and Bullous Pemphigoid: An Updated Review of Clinical Association, Targeted Therapy Exposures and Therapeutic Implications
Source: Biomolecules. 2026 Sep 24;16(10):1391. doi: 10.3390/biom16101391 (PMC13702189; doi:10.3390/biom16101391)
Supplement: Supplementary file 1 [file biomolecules-16-01391-s001.zip › biomolecules-4489626-supplementary Table S1.pdf]

Supplementary Table S1 Complete Search Strategies

All searches were executed on 2 September 2026. The prespecified publication cutoff was 14 April 2026. No language filter was applied at the search stage; English-language eligibility was applied during screening. Counts are the records displayed by each source on the search date and are not inferred from the 41-patient dataset.

Search stream	Source	Complete search string	Limits and handling	Records
Epidemiology	PubMed	("Psoriasis"[Mesh] OR psoriasis[Title/Abstract] OR psoriatic[Title/Abstract]) AND ("Pemphigoid, Bullous"[Mesh] OR "bullous pemphigoid"[Title/Abstract]) AND (epidemiolog*[Title/Abstract] OR prevalence[Title/Abstract] OR incidence[Title/Abstract] OR association[Title/Abstract] OR risk[Title/Abstract] OR cohort[Title/Abstract] OR "case-control"[Title/Abstract] OR "population-based"[Title/Abstract] OR "Mendelian randomization"[Title/Abstract] OR meta-analysis[Publication Type] OR "systematic review"[Title/Abstract]) AND ("1900/01/01"[Date - Publication] : "2026/04/14"[Date - Publication])	1900-01-01 to 2026-04-14	120
Epidemiology	Web of Science Core Collection	TS=((psoriasis OR psoriatic) AND "bullous pemphigoid" AND (epidemiolog* OR prevalence OR incidence OR association OR risk OR cohort OR "case-control" OR "population-based" OR "Mendelian randomization" OR meta-analy* OR "systematic review"))	1900-2026; records after 2026-04-14 excluded during screening	166
Epidemiology	Google Scholar	psoriasis "bullous pemphigoid" epidemiology	1900-2026; relevance order; citations and patents excluded; first 200 results screened	About 9,700; first 200 screened
Mechanisms	PubMed	("Psoriasis"[Mesh] OR psoriasis[Title/Abstract] OR psoriatic[Title/Abstract]) AND ("Pemphigoid, Bullous"[Mesh] OR "bullous pemphigoid"[Title/Abstract]) AND (pathogen*[Title/Abstract] OR mechanis*[Title/Abstract] OR immun*[Title/Abstract] OR "basement membrane zone"[Title/Abstract] OR "epitope spreading"[Title/Abstract] OR BP180[Title/Abstract] OR BP230[Title/Abstract] OR COL17[Title/Abstract] OR "IL-17"[Title/Abstract] OR "IL-23"[Title/Abstract] OR Th17[Title/Abstract] OR Th2[Title/Abstract] OR neutrophil*[Title/Abstract] OR "neutrophil extracellular trap*"[Title/Abstract] OR transcriptom*[Title/Abstract] OR "single-cell"[Title/Abstract]) AND ("1900/01/01"[Date - Publication] : "2026/04/14"[Date - Publication])	1900-01-01 to 2026-04-14	262
Mechanisms	Web of Science Core Collection	TS=((psoriasis OR psoriatic) AND "bullous pemphigoid" AND (pathogen* OR mechanis* OR immun* OR "basement membrane zone" OR "epitope spreading" OR BP180 OR BP230 OR COL17 OR "IL-17" OR "IL-23" OR Th17 OR Th2 OR neutrophil* OR "neutrophil extracellular trap*" OR transcriptom* OR "single-cell"))	1900-2026; records after 2026-04-14 excluded during screening	295
Mechanisms	Google Scholar	psoriasis "bullous pemphigoid" (pathogenesis OR mechanism OR immunology)	1900-2026; relevance order; citations and patents excluded; first 200 results screened	About 13,400; first 200 screened
Targeted therapy case dataset	PubMed	("Psoriasis"[Mesh] OR psoriasis [Title/Abstract] OR psoriatic [Title/Abstract]) AND ("Pemphigoid, Bullous"[Mesh] OR "bullous pemphigoid"[Title/Abstract]) AND ("1900/01/01"[Date - Publication] : "2026/04/14"[Date - Publication])	1900-01-01 to 2026-04-14	459
Targeted therapy case dataset	Web of Science Core Collection	TS=((psoriasis OR psoriatic) AND "bullous pemphigoid")	1900-2026; records after 2026-04-14 excluded during screening	463
Targeted therapy case dataset	Google Scholar	psoriasis "bullous pemphigoid"	1900-2026; relevance order; citations and patents excluded; first 200 results screened	About 16,200; first 200 screened
Targeted therapy case dataset	Reference lists	Backward reference checking of included reports and relevant reviews	Through 2026-04-14	0 additional unique records
