# Supplementary material for: Psoriasis and Bullous Pemphigoid: An Updated Review of Clinical Association, Targeted Therapy Exposures and Therapeutic Implications
Source: Biomolecules. 2026 Sep 24;16(10):1391. doi: 10.3390/biom16101391 (PMC13702189; doi:10.3390/biom16101391)
Supplement: Supplementary file 1 [file biomolecules-16-01391-s001.zip › biomolecules-4489626-supplementary table S2.pdf]

Supplementary Table S2. Published Cases of Concomitant Psoriasis and Bullous Pemphigoid Associated with Targeted Therapy Exposure.

n	Author	Age / Sex	Disease Sequence	Targeted therapy/Class	Descriptive exposure category	Timing to BP onset	Prior phototherapy	Previous BP History	BP diagnostic evidence	Autoantibodies	DIF	Dechallenge /Rechallenge	Other relevant factors	Follow-up	Key Features	Outcome
									**IL-17 inhibitors**							
1	Paschou et al. [42]	72/ M	Psoriasis → BP	Secukinumab (IL-17A)	Temporal association (weak/uncertain)	7 d; ref NR	NR	No	Clinical, histology, DIF, IIF, salt-split skin, ELISA	BP180 (+)	C3 deposition at BMZ	Dechallenge improved; rechallenge NR	Prior pembrolizumab exposure	NR	Tense bullae with erythema	CR; no recurrence reported
2	Oka-mura et al. [43]	56/ M	Long-standing psoriasis → BP	Bimekizumab (IL-17A/F)	Therapeutic use	NA	NR	Yes	Clinical, histology, BP180 ELISA	BP180 (+)	NR	NA	NR	1 year	Bullae/erosions over psoriatic plaques	CR, no relapse
3	Cao et al. [44]	75/ M	Psoriasis → BP	Secukinumab (IL-17A) Dupilumab (IL-4Rα)	Therapeutic use	NA	NR	Yes	Clinical, histology, DIF, BP180 ELISA	BP180 (+)	IgG and C3 at BMZ	NA	Concomitant dupilumab	5 months	Tense blisters with erythematous scaly plaques	CR; no adverse effects reported
4	Lugli et al. [45]	68/F	Pustular psoriasis → BP	Bimekizumab (IL-17A/F)	Temporal association (moderate)	9 wk after 1st dose	NR	No	Clinical, histology, DIF, ELISA	BP180 (+); BP230 (+)	Linear IgG and C3 at BMZ	Dechallenge improved; rechallenge NR	NR	7 weeks	Pustular psoriasis with tense bullae	CR
5	Lugli et al. [45]	64/ M	Psoriasis → BP	Ixekizumab (IL-17A)	Overlapping exposure	204 wk from initiation	NR	No	Clinical, histology, DIF, BP180 ELISA	BP180 (+)	Linear C3 at BMZ	Dechallenge improved; rechallenge NR	No recent treatment change	42 days	Eczema-like eruption	CR

															with new bullae	
6	Wang et al. [46]	49/M	Psoriasis → BP	Secukinumab (IL-17A)	Temporal association (moderate)	1 d after 4th dose	NB-UVB; sessions NR	No	Clinical, histology, DIF, BP180 ELISA	BP180 (+)	Linear IgG at BMZ	Dechallenge improved; rechallenge NR	Prior NB-UVB	NR; no recurrence reported	Scattered tense vesicles with erythematous lesions	CR
7	Wang et al. [47]	71/M	Psoriasis + prior BP → BP relapse	Ixekizumab (IL-17A)	Temporal association (moderate)	4 4 wk from initiation; 1 wk after 2nd dose	NR	Yes	Prior histology; clinical relapse; ELISA	BP180 (+); BP230 (+)	NR	Dechallenge improved; rechallenge NR	NR	NR	Severe pruritus with diffuse erythema and tense bullae	CR
8	Wang et al. [47]	66/M	Long-standing psoriasis → BP	Secukinumab (IL-17A)	Temporal association (moderate)	2 wk from initiation; after 3rd dose	NR	Yes	Prior BP diagnosis; clinical relapse; ELISA	BP180 (+); BP230 (+)	NR	Dechallenge; no rechallenge	NR	29 weeks	Generalized erythema and tense blisters	CR
9	Xiao et al. [49]	63/M	Psoriasis → BP	Ixekizumab (IL-17A)	Therapeutic use	NA	NR	Yes	Clinical, histology, DIF, BP180 ELISA	BP180 (+); BP230 (-)	Linear C3 at BMZ	NA	NR	20 weeks	Blisters on psoriatic and normal skin	CR
10	Drenovska et al. [50]	70/F	Psoriasis → BP	Secukinumab (IL-17A)	Therapeutic use	NA	Phototherapy; type/sessions NR	Yes	Clinical, histology, DIF, IIF, ELISA	BP180 (+); BP230 (+)	Linear IgG and C3 at BMZ	NA	Prior phototherapy and methotrexate	>5 years	Tense bullae on psoriatic and non-lesional skin	CR, no relapse

11	Drenovska et al. [50]	71/F	Psoriasis → BP relapse	Secukinumab (IL-17A)	Temporal association (weak/uncertain)	Within 1 mo from initiation	Quartz lamp; sessions NR	Yes	Clinical, histology, DIF, IIF, ELISA	BP180 (+); BP230 (-)	Linear IgG and C3 at BMZ	Dechallenge improved; rechallenge NR	Methotrexate dose reduction	NR	Generalized tense bullae with erythematous plaques	CR
12	Drenovska et al. [50]	65/M	Psoriasis → BP	Secukinumab (IL-17A)	Therapeutic use	NA	Phototherapy; type/sessions NR	Yes	Clinical, histology, DIF, ELISA	BP180 (+); BP230 (-)	Linear IgG and C3 at BMZ	NA	Recent sun exposure	18 months	Bullae on psoriatic and non-lesional skin	CR, no relapse
13	Yun et al. [51]	68/M	Long-standing psoriasis → BP	Secukinumab (IL-17A)	Therapeutic use	NA	Phototherapy; type/sessions NR	Yes	Clinical, histology, DIF, collagen IV IHC	Anti-BMZ (-)	Linear IgG and C3 at BMZ	NA	NR	7 months	Severe psoriasis with new tense bullae	CR
14	Lu et al. [52]	37/F	Psoriasis → BP	Ixekizumab (IL-17A)	Therapeutic use	NA	NR	Yes	Clinical, histology, DIF, ELISA	BP180 (+); BP230 (+)	Linear C3 at BMZ	NA	NR	1 year	Tense blisters and erosions with scalp involvement	CR; no adverse events
15	Otani et al. [53]	41/M	Pustular psoriasis → BP	Secukinumab (IL-17A)	Therapeutic use	NA	NR	Yes	Clinical, histology, DIF, IIF, salt-split skin, BP180 ELISA	BP180 (+)	Linear IgG and C3 at BMZ	NA	NR	3 years	Pustules with tense bullae and erosions	CR, no relapse

16	Kamata et al. [54]	69/M	Long-standing psoriasis → BP	Secukinumab (IL-17A)	Therapeutic use	NA	NR	Yes	Prior BP diagnosis, BP180 ELISA	BP180 (+)	NR	NA	NR	450 days	Chronic plaques with controlled BP	CR
17	Ho & Tsai [55]	65/F	Psoriasis → BP	Secukinumab (IL-17A)	Temporal association (weak/uncertain)	8 d from initiation; 1 d after 2nd dose	NR	No	Clinical, histology, DIF, salt-split skin, IIF/ELISA	Anti-BMZ (+); BP180 (+)	Linear IgG and C3 at BMZ	Dechallenge improved; rechallenge negative	NR	NR	Hemorrhagic bullae over psoriatic plaques	CR
									**IL-12/23 and IL-23 inhibitors**							
1	Xi et al. [56]	78/F	Psoriasis → BP	Guselkumab (IL-23p19)	Temporal association (moderate)	2 wk after 1st dose	NR	No	Clinical and histology	BP180 (-); BP230 (-)	Not performed	Dechallenge improved; rechallenge NR	No recent medication change	NR	Generalized tense bullae	CR within 1 month; no recurrence reported
2	Sugai et al. [57]	74/M	Psoriasis → prior BP	Risankizumab (IL-23p19)	Therapeutic use	NA	NR	Yes	Clinical, histology, DIF, BP180 ELISA	BP180 (+)	Positive; details NR	NA	NR	2.3 years	Serologically active BP without bullae	CR, no relapse
3	Sugai et al. [57]	56/F	Psoriasis → BP	Guselkumab (IL-23p19)	Therapeutic use	NA	NR	Yes	Clinical, histology, DIF, BP180 C-terminal immunoblotting	BP180 (-); BP180 C-terminal IB (+); anti-p200 (-)	Linear IgG and C3 at BMZ	NA	NR	5 years	Bullae and erosions	CR, no relapse

4	Rodríguez-Cuadrado et al. [58]	48/M	Long-standing psoriasis → BP	Guselkumab (IL-23p19)	Therapeutic use	NA	No	Yes	Clinical, histology, DIF, ELISA	BP180 (+); BP230 (+)	Linear IgG and C3 at BMZ	NA	Multiple prior systemic therapies	2 years	Tense bullae and erosions	CR, no relapse
5	Cole & Amber [59]	68/F	BP + psoriasis	Ustekinumab (IL-12/23p40)	Therapeutic use	NA	NR	Yes	Prior BP diagnosis	BP230 (+)	NR	Interruption flare; re-initiation improved	Prednisone and NB-UVB	2 years	Pruritic bullae	CR, no relapse
6	Cole & Amber [59]	71/M	Prior BP → psoriasis/BP coexistence	Tildrakizumab (IL-23p19)	Therapeutic use	NA	NR	Yes	Prior BP diagnosis	BP180 (+); BP230 (+)	NR	NA	Cyclosporine taper	NR	Erythrodermic psoriasis with concomitant BP	CR
7	Kong et al. [60]	71/M	Psoriasis → BP	Ustekinumab (IL-12/23p40)	Temporal association (strong)	2 wk after 1st dose; recurrence 1 wk after 2nd dose	NR	No before first exposure	Clinical, histology, DIF, ELISA	BP180 (+); BP230 (+)	Linear IgG at BMZ	Dechallenge improved; rechallenge positive	NR	2 months after discontinuation	Pruritic tense bullae on upper limbs	CR
8	Fukada et al. [61]	80/F	Psoriasis → BP	Risankizumab (IL-23p19)	Temporal association (moderate)	2 d after 6th dose	NR	No	Clinical, histology, DIF, salt-split IIF, ELISA	BP180 (+); BP230 (-)	Linear IgG and C3 at BMZ	Dechallenge improved; rechallenge NR	NR	NR	Pruritic erythema, tense bullae, and erosions	CR
9	Burlando et al. [62]	76/M	Psoriasis → BP	Guselkumab (IL-23p19)	Temporal association (weak/uncertain)	4 wk from initiation	NR	No	Clinical, histology, DIF, ELISA	BP180 (+); BP230 (+)	Linear IgG and C3	Dechallenge improved; rechallenge NR	Recent switch from ustekinumab	4 months	Generalized tense bullae and erosions	CR, no relapse

											at BMZ					
10	Querol-Cisneros et al. [18]	76/F	Long-standing psoriasis → BP	Ustekinumab (IL-12/23p40)	Temporal association (moderate)	2 mo from initiation	NR	No	Clinical, histology, DIF, IIF, salt-split skin	Anti-BMZ IgG (+) by IIF; specific autoantibodies not detected	Linear IgG and C3 at BMZ	Dechallenge improved; rechallenge NR	NR	2 years	Pruritic plaques with tense blisters	CR, no relapse
11	Marin et al. [63]	75/F	Long-standing psoriasis → BP	Ustekinumab (IL-12/23p40)	Temporal association (moderate)	10 mo from initiation; 5 d after 5th dose	NR	No	Clinical, histology, split-skin DIF/IIF	BP180 (-); anti-epidermal autoantibodies (+)	Linear IgG and C3 at BMZ	Dechallenge improved; rechallenge not performed	Multiple prior systemic therapies	3 months	Pruritic erythematous plaques with tense bullae	CR
12	Loget et al. [64]	88/F	Psoriasis → relapsing BP	Ustekinumab (IL-12/23p40)	Therapeutic use	NA	NR	Yes	Clinical, histology, DIF, salt-split IIF, ELISA	BP180 (+); BP230 (-)	Linear C3 at BMZ	NA	NR	2 years	Disseminated plaques with frequent blisters	CR, no relapse
13	Nakayama et al. [65]	63/M	Psoriatic onycho-pachydermo-periostitis → BP	Ustekinumab (IL-12/23p40)	Temporal association (weak/uncertain)	21 mo from initiation	NR	No	Clinical, histology, DIF, salt-split IIF, ELISA	BP180 (+)	Linear IgG, IgM and C3 at BMZ	Dechallenge improved; rechallenge NR	NR	NR	Diffuse erythema with pruritic blisters and erosions	CR

**TNF-α inhibitors**																
1	Gambichler et al. [17]	67/F	Long-standing psoriasis → BP	Adalimumab (TNF-α)	Overlapping exposure	NR	NR	Yes	Clinical, histology, DIF, ELISA	BP180 (+); BP230 (+)	Linear IgG at BMZ	NR	Multiple prior systemic therapies	NR	Widespread bullae and erosions	PR
2	Tirado-Sánchez & Bonifaz [66]	45/M	Long-standing psoriasis → BP	Adalimumab (TNF-α)	Temporal association (strong)	8 wk from initiation; after 3rd maintenance dose	NR	No	Clinical, histology, DIF	NR	Linear IgG and C3 at BMZ	Dechallenge improved; rechallenge positive	NR	NR	Multiple pruritic tense blisters	CR
3	Saraceno et al. [34]	55/M	Long-standing psoriasis → BP	Etanercept (TNF-α); Rituximab (CD20)	Therapeutic use	NA	UVB; sessions NR	Yes	Clinical, histology, DIF, BP180 ELISA	BP180 (+)	Linear IgG and C3 at BMZ	NA	Losartan exposure	NR	Generalized bullae and erosions	CR
**JAK inhibitors**																
1	Hu et al. [67]	58/M	Long-standing psoriasis → BP	Abrocitinib (JAK1)	Therapeutic use	NA	NR	Yes	Clinical, histology, BP180 ELISA	BP180 (+)	NR	NA	NR	8 months	Pruritic tense bullae with psoriatic plaques on extremities	CR, no relapse
2	Su et al. [68]	66/M	Prior psoriasis → BP; psoriasis flare during BP treatment	Upadacitinib (JAK1)	Therapeutic use	NA	NR	Yes	Clinical, histology, DIF, ELISA	Anti-BMZ (+); BP180 (±);	Linear C3 at BMZ	NA	Steroid taper and dupilumab	10 weeks	BP with psoriasis relapse	CR

										BP230 (-);						
3	Li et al. [69]	33/M	Psoriasis → BP	Tofacitinib (JAK1/3)	Therapeutic use	NA	NR	Yes	Clinical, histology, DIF, salt-split IIF, BP180 ELISA	BP180 (+)	Linear IgG and C3 at BMZ	NA	NR	48 weeks	Tense bullae and erosions on forehead and upper limbs	CR, no relapse
4	Xiao et al. [70]	83/M	Plaque psoriasis → BP	Baricitinib (JAK1/2)	Therapeutic use	NA	NR	Yes	Clinical, BP180 ELISA, salt-split study	BP180 (+)	NR	NA	NR	24 weeks	Generalized tense blisters and erosions with pruritus	CR; no adverse effects
									**PDE4 inhibitors**							
1	Kumagai et al. [71]	85/M	Long-standing psoriasis → BP	Apremilast (PDE4)	Therapeutic use	NA	NR	Yes	Clinical, histology, DIF, BP180 ELISA	BP180 (+)	Linear IgG and C3 at BMZ	NA	Concomitant minocycline/ nicotinic acid	3 weeks	Generalized tense blisters and erythema	CR
2	Kitayama et al. [72]	65/M	Psoriasis → BP	Apremilast (PDE4)	Therapeutic use	NA	NR	Yes	Clinical, histology, DIF, salt-split IIF, BP180 ELISA	BP180 (+)	Linear IgG and C3 at BMZ	NA	NR	36 weeks	Erythematous plaques with overlying bullae and erosions	CR, no relapse

							**Other targeted agents**									
1	Teshima et al. [73]	75/M	Psoriasis + BP → GPP	Spesolimab (IL-36R)	Therapeutic use	NA	NR	Yes	Prior BP diagnosis	NR	NR	NA	Systemic corticosteroid exposure	NR	New pustules over plaques with BP background	CR, no relapse
2	Liu et al. [74]	73/M	Long-standing psoriasis → BP	Dupilumab (IL-4Rα)	Therapeutic use	NA	NR	Yes	Clinical, histology, DIF, ELISA	BP180 (+); BP230 (+)	Linear IgG and C3 at BMZ	NA	Chinese herbal compounds	6 months	Generalized tense blisters with oral erosions	PR, no relapse

* Abbreviations: anti-BMZ, anti-basement membrane zone; BMZ, basement membrane zone; BP, bullous pemphigoid; BP180, bullous pemphigoid antigen 180; BP230, bullous pemphigoid antigen 230; C3, complement component 3; CD20, cluster of differentiation 20; CR, complete remission; DIF, direct immunofluorescence; ELISA, enzyme-linked immunosorbent assay; F, female; GPP, generalized pustular psoriasis; IB, immunoblotting; IgG, immunoglobulin G; IgM, immunoglobulin M; IHC, immunohistochemistry; IIF, indirect immunofluorescence; IL, interleukin; IL-4Rα, interleukin-4 receptor alpha; IL-36R, interleukin-36 receptor; JAK, Janus kinase; M, male; NA, not applicable; NB-UVB, narrowband ultraviolet B; NC16A, non-collagenous 16A domain of BP180; NR, not reported; p200, 200-kDa dermal antigen; PDE4, phosphodiesterase 4; PR, partial remission; TNF-α, tumor necrosis factor alpha. Other relevant factors include alternative potential causes or concomitant treatments potentially relevant to BP onset or disease course. 'Temporal association' denotes cases in which BP onset or exacerbation occurred after targeted therapy exposure and does not imply established causality. For these cases, causal support was further graded as strong, moderate, or weak/uncertain according to the predefined criteria described in the Methods. 'Therapeutic use' denotes cases in which the targeted therapy was administered primarily for disease control. 'Overlapping exposure' denotes treatment exposure without a sufficiently clear temporal relationship to BP onset or exacerbation. These categories are used solely for descriptive organization and do not constitute formal causality assessments. Timing to BP onset is reported according to the reference point provided in the original publication; values with different reference points are not directly comparable. Prior phototherapy refers to phototherapy before the relevant BP onset or exacerbation; modality and number of sessions

are reported when available. Objective causality-related features, including timing to BP onset, previous BP history, dechallenge, rechallenge, diagnostic evidence, and alternative or concomitant factors, are reported separately when available. Missing information is designated as NR and was not inferred.
